# Co-expression of Ubiquitin gene and capsid protein gene enhances the potency of DNA immunization of PCV2 in mice

**DOI:** 10.1186/1743-422X-8-264

**Published:** 2011-05-30

**Authors:** Fang Fu, Xuesong Li, Yuekun Lang, Yuju Yang, Guangzhi Tong, Guoxin Li, Yanjun Zhou, Xi Li

**Affiliations:** 1 Division of Swine Infectious Diseases, State Key Laboratory of Veterinary Biotechnology, Harbin Veterinary Research Institute, Chinese Academy of Agricultural Sciences No. 427 Maduan St., Nangang District, Harbin, 150001, China; 2 Department of Science, Harbin University No. 9 Xuefu Street, Harbin, Heilongjiang, 150086, China; 3 Shanghai Veterinary Research Institute, Chinese Academy of Agricultural Sciences, No. 518 Ziyue Road, Minhang District, Shanghai 200241, China

**Keywords:** PCV2, DNA immunization, Cap, Ubiquitin

## Abstract

A recombinant plasmid that co-expressed ubiquitin and porcine circovirus type 2 (PCV2) virus capsid protein (Cap), denoted as pc-Ub-Cap, and a plasmid encoding PCV2 virus Cap alone, denoted as pc-Cap, were transfected into 293T cells. Indirect immunofluorescence (IIF) and confocal microscopy were performed to measure the cellular expression of Cap. Three groups of mice were then vaccinated once every three weeks for a total of three doses with pc-Ub-Cap, pc-Cap or the empty vector pCAGGS, followed by challenging all mice intraperitoneally with 0.5 mL 10^6.5 ^TCID_50_/mL PCV2. To characterize the protective immune response against PCV2 infection in mice, assays of antibody titer (including different IgG isotypes), flow cytometric analysis (FCM), lymphocyte proliferation, cytokine production and viremia were evaluated. The results showed that pc-Ub-Cap and pc-Cap were efficiently expressed in 293T cells. However, pc-Ub-Cap-vaccinated animals had a significantly higher level of Cap-specific antibody and induced a stronger Th1 type cellular immune response than did pc-Cap-vaccinated animals, suggesting that ubiquitin conjugation improved both the cellular and humoral immune responses. Additionally, viral replication in blood was lower in the pc-Ub-Cap-vaccinated group than in the pc-Cap and empty vector groups, suggesting that the protective immunity induced by pc-Ub-Cap is superior to that induced by pc-Cap.

## 1. Background

Porcine circovirus type 2 (PCV2) is a small, non-enveloped, single-stranded, circular DNA virus with a 1767 nt or 1768 nt ambisense genome [1] that contains at least two major open reading frames (ORFs). ORF1 encodes the replication proteins (Rep and Rep') involved in virus replication and ORF2 encodes the capsid protein (Cap) [2,3]. Cap, a protein associated with the development of neutralizing antibodies and antibody protection [4,5], has been a leading target for designing new vaccines against PCV2 infection.

Immunologic potential of a DNA vaccine encoding the PCV2 Cap in mice was first investigated by Kamstrup, et al. [6]. DNA vaccines may be capable of inducing immunity regardless of maternally derived antibodies [7-9] and they have induced protective cellular and humoral immunity in preclinical models of infectious diseases. However, DNA vaccine applications are limited due to problems related to delivery, species of the immunized animals and degradation of plasmid DNA [10], resulting in modest cellular and humoral immune responses [11]. To compensate for these limitations, numerous studies have explored methods to improve immune responses induced by DNA immunization by optimizing plasmid design, vaccine delivery systems and adjuvants [12]. Adjuvants are of particular interest because they may enhance DNA delivery and increase the magnitude and duration of plasmid DNA expression [13]. Molecular adjuvants, such as co-stimulatory chemokines and cytokines, have been used previously in conjunction with DNA vaccines and have served as immune modulators [14].

Ubiquitin, a 76-amino-acid peptide found in the cytoplasm of eukaryotic cells, is normally involved in controlling intracellular protein turnover [15] and was reported to enhance DNA vaccine responses against antigens in the adjuvant setting. Ubiquitinated proteins targeted to the proteasome system [16] are processed and presented through the major histocompatibility complex (MHC) class I pathway to stimulate differentiation and clonal expansion of MHC class I-restricted T cells, which are typically CD8^+^, cytotoxic T cells [17-19]. This strategy enhances proteasome-dependent degradation of endogenously synthesized antigens and results in an increased cell-mediated response against the conjugated antigen in vivo [20-22]. Tuberculosis and influenza virus [23,24] DNA vaccines using ubiquitin to enhance the immune response showed better results compared to DNA vaccine alone.

In this study, BALB/c mice were vaccinated with pc-Ub-Cap and pc-Cap to investigate whether ubiquitin conjugation to ORF2 would enhance the immune response. In addition, pc-Ub-Cap vaccination was compared with pc-Cap vaccination to assess if pc-Ub-Cap provided better protection against PCV2. The results demonstrated that ubiquitin conjugation improved both the cellular and humoral immune responses in PCV2 DNA vaccinated animals and that the protective immunity induced by pc-Ub-Cap is superior to that induced by pc-Cap.

## 2. Methods

### 2.1 Virus, cells, mice and plasmids

The virulent PCV2 isolate, 871 (no. EU420015), was originally isolated from pigs with naturally occurring postweaning multisystemic wasting syndrome (PMWS) and serially passaged 32 times in PK-15 cells. 293T cells used for transfection were maintained at Harbin Veterinary Research Institute of China and grown in minimal essential medium (Gibco) supplemented with penicillin, streptomycin and 10% heat-inactivated fetal bovine serum (FBS; Gibco). Eight-week-old female BALB/c mice were purchased from Harbin Veterinary Research Institute of Chinese Academy of Agricultural Science and raised in automatic, extrusion-independent venting isolation cages. Animal maintenance and experimental protocols were approved by the Animal Experiment Ethics Committee of the authors' institute.

The recombinant plasmids, pMD18-T-ORF2 and pMD18-T-ubiquitin, were generated using ORF2 and ubiquitin fragments inserted into pMD18-T and maintained at Harbin Veterinary Research Institute of China. The ORF2 gene coding wild-type Cap was amplified from the total DNA of PCV2 by polymerase chain reaction (PCR). The ubiquitin gene was synthesized based on the pig ubiquitin amino acid sequence with Gly76 changed to Arg76 (no. M18159). The Kozak sequence, GCCACC, served as the upstream start codon for both fragments.

### 2.2. Construction of the eukaryotic expression plasmids

All expression plasmids were constructed using pCAGGS (a eukaryotic expression vector kindly provided by Dr. Zhigao Bu of the Harbin Veterinary Research Institute) as a vector. Primers used for PCR amplification are listed in Table 1. The entire ORF2 was amplified from the recombinant plasmid pMD18-T-ORF2 using the primer pair FW/RV. The PCR product digested with *Xho *I, *Bgl *II was subcloned into the mammalian expression vector, pCAGGS (Promega), to construct the recombinant expression plasmid, pc-Cap. To generate the coexpression plasmid, a 790 bp DNA fragment with a linker encoding the full length Cap protein and a 270 bp DNA fragment with a linker encoding the ubiquitin gene, were amplified by PCR using primer pairs fw1/rv1 and fw2/rv2, respectively. The PCR products were used as a template to construct the Ub-Cap fusion gene. This fusion gene was generated by linking ORF2 downstream of ubiquitin and was amplified using the fw1/rv2 primer pair. The PCR product was digested with *Sac *I, *Xho *I and subcloned into the expression vector, pCAGGS, to construct the recombinant expression plasmid, pc-Ub-Cap. Finally, the plasmids were purified using an EndoFree Plasmid Giga kit column (Qiagen) for use as a DNA vaccine.

**Table 1:** Oligonucleotide primers used in this study

Gene	Primers	Sequences of primers(5'→3')	Length	Anneal tem
ORF2	Fw	AAT*CTCGAG*GCCACCATGACGTATCCAAGG	717 bp	56°C
				
	Rv	ATT*AGATCT*TTATTCATTAAGGGTTAAGTG		

ub-linker	fw1	gga *gagctc*gccaccatgcagatcttcgtg	270 bp	56°C
				
	rv1	gccgccgccgctgccgccgccgccgctgccgccgccgcctcttcccctcaagcg		

linker-ORF2	fw2	ggcggcggcggcagcggcggcggcggcagcggcggcggcATGACGTATCCAAGG	790 bp	56°C
				
	rv2	*ctcgag *TTATTCATTAAGGGTTAAGTGGGGGG		

U-ORF2	fw1	gga *gagctc *gccaccatgcagatcttcgtg	1030 bp	56°C
				
	rv2	*ctcgag *TTATTCATTAAGGGTTAAGTGGGGGG		

Note: Italic denotes restriction enzyme site; underline denotes linker; TTA denotes stop.

### 2.3. IIF and confocal microscopy

At 48 h post-transfection, 293T cell were fixed with absolute methanol for 5 min and processed for an IIF assay using a monoclonal antibody (mAb) against the Cap protein of PCV2 (1:200) [25], followed by application of FITC-conjugated, goat-anti-mouse IgG (1:100) (Sigma). Fluorescent images were examined under an inverted fluorescence microscope (Olympus IX70).

Forty-eight hours post-transfection, cells were fixed using 4% paraformaldehyde for 20 min at room temperature and then incubated separately with PCV2 Cap-specific mAb (1:100) and FITC-conjugated, goat-anti-mouse IgG (1:64). After washing with PBS-T (1×PBS, 0.5% Tween 20), the cells were stained with 100 μg/mL PI for 15 min at room temperature. The cells were washed three times with PBS-T and then analyzed with a Leica confocal microscope using the Leica confocal software (TCS SP5 version).

### 2.4. In vivo immunization and challenge

Seventy-five mice were randomized into three groups of 25 mice each. Two groups of 25 mice were immunized with pc-Cap and pc-Ub-Cap, respectively, and the third group of twenty-five mice was immunized with the empty vector, pCAGGS. For immunization, 100 μg of plasmid DNA was injected into the quadriceps femoris muscles once every three weeks for a total of three doses. Serum samples for Enzyme-Linked Immunosorbent Assay (ELISA) analysis were collected from the tail vein every two weeks after immunization until the end of the experiment. Six weeks after the final injection, five mice from each group were euthanized and tissues were harvested for flow cytometric analysis (FCM), lymphocyte proliferation assays and cytokine assays by ELISA, including IFN-γ and IL-2. At 18 weeks p.i., the remaining twenty mice from each group were challenged intraperitoneally with 0.5 mL PCV2 inoculum (10^6.5 ^TCID_50_/mL). Five mice from each group were euthanized at two, four, six and eight weeks p.c., and whole blood was collected for quantification of the PCV2 viral load by real-time PCR as described previously [26].

### 2.5. Detection of Cap-specific IgG, IgG1 and IgG2a antibodies

An endpoint ELISA was performed to assess the titers of total IgG, IgG1 and IgG2a antibodies against PCV2 Cap protein. The recombinant Cap protein with deleted nuclear localization signal (deletion of the 41, N-terminal amino acid residues was used as the coating antigen [27]. Briefly, 96-well plates (Jet) were coated overnight at 4°C with 0.1 mL of purified recombinant antigen (5 mg/mL) in a coating solution (bicarbonate buffer, pH 9.6). The plates were then blocked with 5% skim milk and serum samples were applied in 0.1 mL of serial two-fold dilutions, starting with a 1:64 dilution. The plates were then incubated at 37°C for 40 min. The bound antibodies were detected by horseradish peroxidase-conjugated goat anti-mouse IgG, IgG1 or IgG2a antibody (diluted 1:6,000; Southern Biotechnology Associates). The substrate, 3, 3-, 5-, 5-tetramethylbenzidine (TMB Sigma) was used to visualize the reaction. The results were expressed as the ratio of OD_450 _produced by the serum samples compared with negative control serum. Ratio values higher than 2.1 were considered to be positive for antibody, and antibody titers were then defined as the reciprocal of the highest dilution of sample for which the ratio value was higher than 2.1. The datas are presented as the log_2 _value of each titer.

### 2.6. Lymphocyte proliferation assay

Six weeks after the final injection, splenocytes were isolated as described previously by Kang, et al. [28]. Splenocytes were cultured in RPMI-1640 with 10% FBS containing penicillin (100 units/ml) and streptomycin (100 units/mL) in triplicate at a density of 1 × 10^5 ^cells/well (100 μl total volume) in 96-well plates. The cultures were stimulated for 18 h with either 20 μl 10^6.5 ^TCID_50_/mL inactivated PCV2 as the specific antigen or with no antigen as a negative control. After incubation at 37°C in 5% CO_2 _for 96 h, 20 μl of Cell Counting Kit-8 (Beyotime Biotech Co., Ltd) was added to each well and the cells were incubated for 1 h. At the end of the incubation, the plates were measured at 450 nm. The stimulation index (SI) was calculated as the ratio of the average OD value of wells containing antigen-stimulated cells to the average OD value of wells containing cells cultured with medium alone.

### 2.7. Flow cytometric analysis (FCM)

Flow cytometric analysis was performed to determine the percentage of CD4+T and CD8+T cells. All conditions were repeated in triplicate. Splenocytes (10^6 ^cells) were stimulated for 18 h with 20 μl of 10^6.5 ^TCID_50_/mL inactivated PCV2 as the specific antigen or with no antigen as a negative control. After culturing for 18 h at 37°C, splenocytes were resuspended in 25 μl PBS and incubated with the following antibodies at a concentration of 0.075 μg, 0.05 μg and 0.1 μg per 10^6 ^cells: 25 μl diluted (1:200), FITC-conjugated, anti-mouse CD4 (L3T4; BD Biosciences); Percp-conjugated anti-mouse CD3 (CD3ε chain; BD Biosciences) mAb; and PE-conjugated anti-mouse CD8a (Ly-2; BD Biosciences) mAb. After 30 min incubation, the cells were washed and analyzed with a FACS Calibur cytometer using CellQuest software (BD Biosciences). A minimum of 10^4 ^cells were analyzed for each sample.

### 2.8. Cytokine assay

Culture supernatants of lymphocyte were removed aseptically from 96 wells after incubation, as in the lymphocyte proliferation assay. Mouse IFN-γ and IL-2 were quantified in the culture supernatants of splenocytes by ELISA kit (Beijing 4A Biotech Co., Ltd) according to manufacturer instructions. The cytokine assay was performed as follows: 100 μL diluted standard or sample was added to each well, followed by 50 μl biotin-conjugated anti-cytokine antibody. The plate was covered with aluminum foil and incubated for 120 min at room temperature. The plate was then washed four times with 350 μl of wash buffer, and 100 μl of enzyme conjugate working solution was added to each well and allowed to incubate at room temperature for 30 min. The plate was washed four more times, and 100 μl of chromogenic substrate was incubated in each well for 20 min at room temperature to develop the colorimetric reaction. Color development was stopped with 100 μL stop buffer. The plate was read immediately at 450 nm on the Universal Microplate Reader ELx800 (Bio-Tek Instruments, Inc., Winooski, VT, USA). A standard curve for each cytokine was obtained using the standard protein provided by the manufacturer.

### 2.9. Quantitative real-time PCR for evaluation of viremia

At two, four, six and eight weeks p.c., DNA was extracted from 500 μl of whole blood by using the DNeasy Blood & Tissue kit (Qiagen, Chatsworth, CA, USA). Viral DNA levels in samples collected after PCV2 challenge were determined using a quantitative, real-time PCR as described previously [26]. A forward (5'-ATTAGTCTTCCAATCACGCTTCTG-3') and a reverse (5'-CTTGTTGGAGAGCGGGAGTC-3') primer were used to amplify a 135 bp fragment from the ORF2 of PCV2. SYBR real-time PCR was performed using the BIO-RAD iQ5 (Corbett, Sydney, Australia) and 12.5 μL of SYBR^® ^Premix Ex Taq™ (Perfect Real Time, TaKaRa, Dalian, China) was used in each reaction. Serial dilutions of plasmid pORF2 were used to obtain a standard curve. The numbers of virus copies for each sample is presented as the mean value of duplicate reactions. The detection limit of this assay was 10^2 ^copies/mL.

### 2.10. Statistical analysis

One-way analysis of variance (ANOVA) was used to determine statistical differences in humoral and cellular immune responses between groups. Differences where p < 0.05 were considered significant. Statistical analysis was performed using SPSS version 12.0 (SPSS) and Statistical Analysis System (SAS) for windows 6.12 (SAS Institute Inc., Cary, NC, USA).

## 3. Results

### 3.1. Expression levels of recombinant plasmids

**Figure 1:**
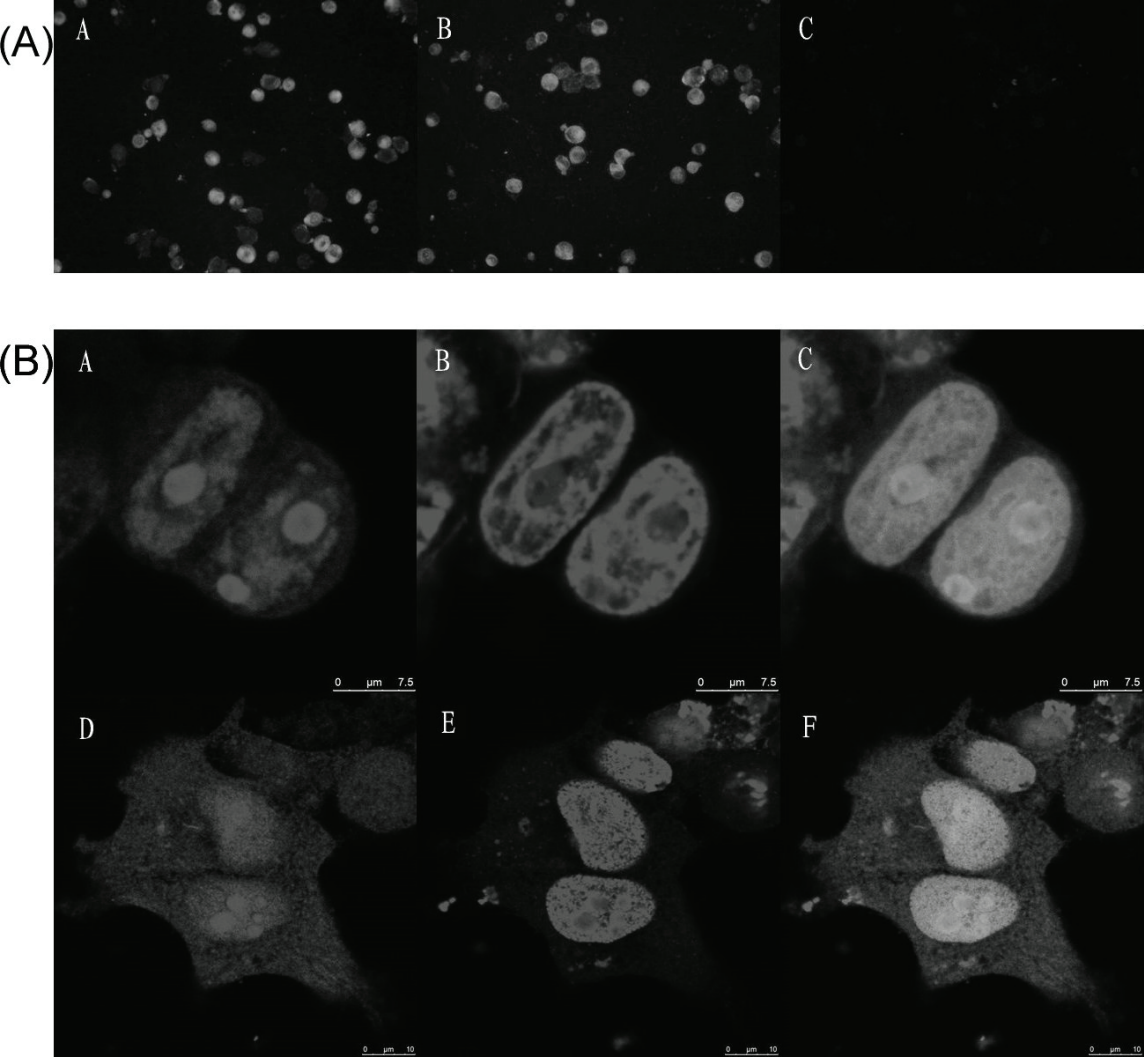
Expression of the Cap protein in vitro. (A) Indirect immunofluorescence detection of Cap on 293T cells. 293T cells were previously transfected with 1 μg of different recombinant plasmids or the empty vector, in a 12-well plate. Forty-eight hours post-transfection, cells were fixed with cold acetone, treated with anti-Cap monoclonal antibody (mAb), and stained with FITC conjugated secondary antibody. Green: Cap protein-positive 293T cells; dark: negative cells. A was transfected with pc-Cap, B with pc-Ub-Cap and C with vector. (B) 293T cells were transiently transfected with pc-Cap and pc-Ub-Cap. 48 h post transfection cells were fixed and the distribution of recombinant proteins was analyzed by confocal microscopy. Localization of recombinant product in 293T cells. (A and D) Incubated with of PCV2 Cap specific mAb followed by incubation with a secondary antibody conjugated with FITC. (B and E) staining of the nucleic lamina with PI. (C and F) Merge of A and B, D and E. Green channel (left pannel), red channel (middle pannel) and merge (right pannel). The bar represents 7.5 μm.

To investigate whether the Cap protein is expressed in 293T cells transfected with pc-Cap and pc-Ub-Cap, IIF and confocal microscopy were performed at 48 h post-transfection. The IIF assay showed that 293T cells transfected with pc-Cap and pc-Ub-Cap were recognized by mAb to Cap at 48 h post-transfection, while the pCAGGS infected 293T cells were not recognized by the specific mAb (Figure 1A). Confocal microscopy analysis showed that the expressed Cap protein was localized in the nuclei of cells (Figure 1B).

### 3.2. Total IgG and isotypes of serum antibody against PCV2 Cap protein

**Figure 2:**
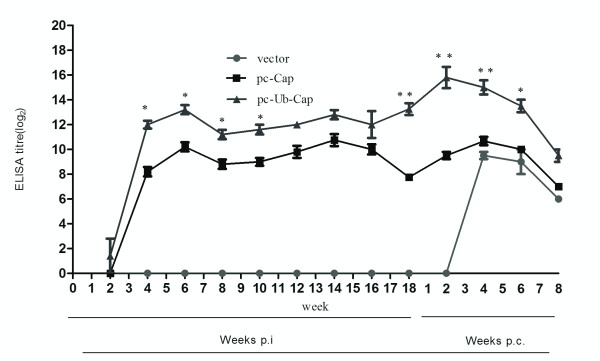
**Kinetics of total IgG production against PCV2 Cap protein at various times p.i**. The results are shown as means ± SD (n = 5). Significant differences between the vaccines are indicated: **, P < 0.01; *, P < 0.05.

To evaluate for seroconversion of the mice, an indirect ELISA was performed to detect PCV2 Cap protein specific antibodies. As shown in Figure 2, the titers for total IgG against PCV2 Cap protein in the pc-Ub-Cap group seroconverted at two weeks post immunization (p.i.), while those in the pc-Cap vaccine groups seroconverted at four weeks p.i. Four weeks after primary immunization, the antibody titer reached a detectable level in all vaccinated groups except for the group immunized with the vector. A further increase in antibody levels was observed at six weeks after primary immunization. The pc-Ub-Cap protocol induced higher IgG titers with peaks of 13.2 ± 0.837 log_2 _at 12 weeks p.i. compared with titers from the pc-Cap (10.2 ± 0.837 log_2_) vaccines after the same amount of time. This difference was statistically significant at eight weeks (*P *< 0.05), 10 weeks and 12 weeks (*P *< 0.01) after primary immunization. Anti-Cap antibodies developed earlier and with higher titers in the pc-Ub-Cap compared to the pc-Cap-immunized group. After challenge, the antibody levels in both vaccine groups increased sharply compared to the control group, though the antibody values with pc-Ub-Cap were remarkably higher than with pc-Cap. Throughout the testing period, Cap antibody levels in pc-Ub-Cap-immunized mice were higher than in the pc-Cap-immunized mice.

**Figure 3:**
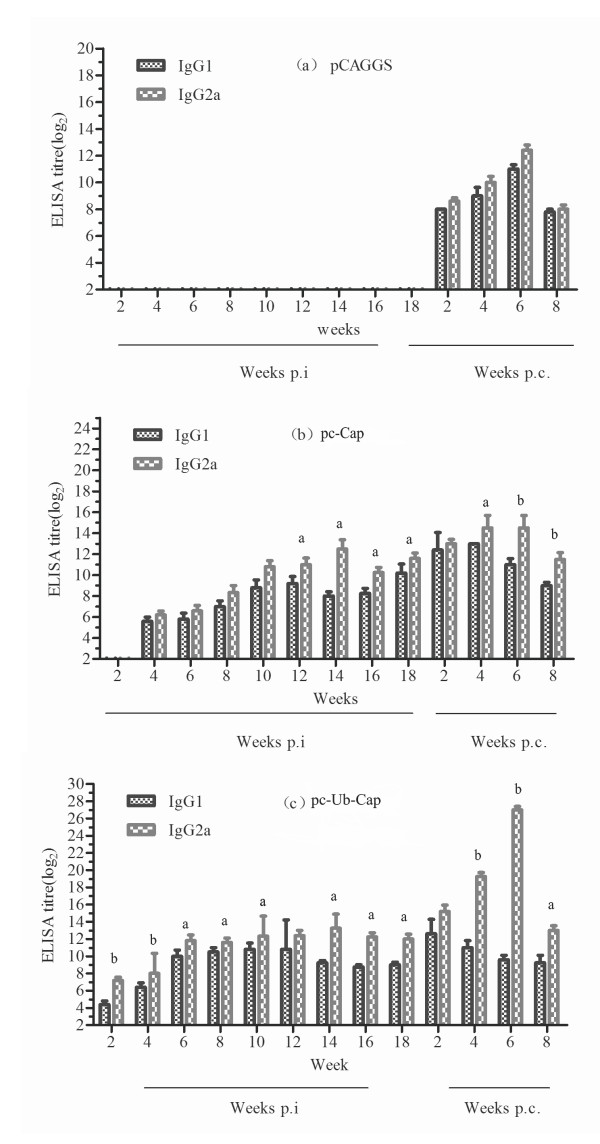
**Cap-specific IgG isotypes induced in the pc-Cap(b) and pc-Ub-Cap(c), and control (a)groups**. The results are shown as means ± SD (n = 5). The letters 'a' and 'b' above columns indicate significantly higher IgG isotype with values of P < 0.05 and P < 0.01, respectively, at the same time in a group. Mice were challenged at 18 weeks p.i.

We evaluated the IgG1/IgG2a isotypes against PCV2 Cap protein to determine the antibody response profile induced by pc-Cap and pc-Ub-Cap. Both pc-Cap and pc-Ub-Cap immunized mice had significantly higher levels of IgG2a than IgG1. The pc-Cap plasmid stimulated significantly higher IgG2a antibodies than IgG1 from 12 to 18 weeks p.i. (P < 0.01), with a peak IgG2a titer of 12.4 ± 1.517 log_2 _at 14 weeks (Figure 3b). Similarly, the pc-Ub-Cap plasmid stimulated significantly higher IgG2a antibodies than IgG1 from 2 to 18 weeks p.i. (two to four weeks p.i. P < 0.01; six to ten weeks and 14 to 18 weeks p.i., P < 0.05), with a peak IgG2a titer of 13.2 ± 1.483 log_2 _at 14 weeks (Figure 3c). Strikingly, mice immunized with pc-Cap and pc-Ub-Cap produced detectable levels of Cap-specific IgG2a antibodies. Though both pc-Ub-Cap and pc-Cap resulting in high IgG2a levels, immunization with pc-Ub-Cap DNA vaccine did still result in significantly higher level of IgG2a antibodies than the pc-Cap vaccine in the time period from two to eight weeks p.i. (p < 0.01).

### 3.3. Enhanced T cell proliferation in response to pc-Ub-Cap

To measure the T cell proliferative response, splenocytes from mice were isolated and restimulated *in vitro *with inactivated PCV2 six weeks after the last immunization. As shown in Table 2 mice immunized with pc-Ub-Cap had strong stimulation of lymphocytes sensitized PCV2 and reached the highest proliferative activity compared to mice immunized with the vector. Notably, both pc-Cap and pc-Ub-Cap elicited a proliferative response that was absent in mice administered the vector alone. However, splenocytes from mice immunized with pc-Ub-Cap induced significantly stronger T cell proliferation responses to the viral antigen than those from mice immunized with pc-Cap. The pc-Ub-Cap plasmid elicited significantly higher cell proliferation than the pc-Cap (p < 0.05).

**Table 2:** PCV2 Cap-specific lymphoroliferative response and FCM analysis of mice splenocytes

Group	SI	stimulation of inactivated PCV2(mean mice splenocytes ± SD)	
		**CD4^+ ^cells(%)**	**CD8^+ ^cells(%)**

Vector	1.00 ± 0.00^c^	9.42 ± 0.75^c^	4.34 ± 0.28^c^
pc-Cap	1.29 ± 0.107^b^	16.6 ± 1.35^b^	5.33 ± 0.23^b^
pc-Ub-Cap	1.73 ± 0.098^a^	23.02 ± 1.98^a^	7.47 ± 0.36^a^

Shared letters above columns indicate no significant difference (P > 0.05), whilst different letters indicate significant differences among the groups (P < 0.05) (n = 5)

### 3.4. Cap-induced cellular immune responses

The proportion of CD4^+ ^T cells and CD8^+ ^T cells in the stimulated splenocytes were determined by FCM. As shown in Table 2 significant upregulation of CD4^+ ^T cells and CD8^+ ^T cells was elicited in all of the vaccine groups compared with the control group (P < 0.05). With regard to the expression of CD4^+ ^T cells and CD8^+ ^T cells, the pc-Ub-Cap group showed significant increases (P < 0.05) when compared with the pc-Cap group.

### 3.5. Ub-Cap constructs promote Th1 type cellular immune responses

**Figure 4:**
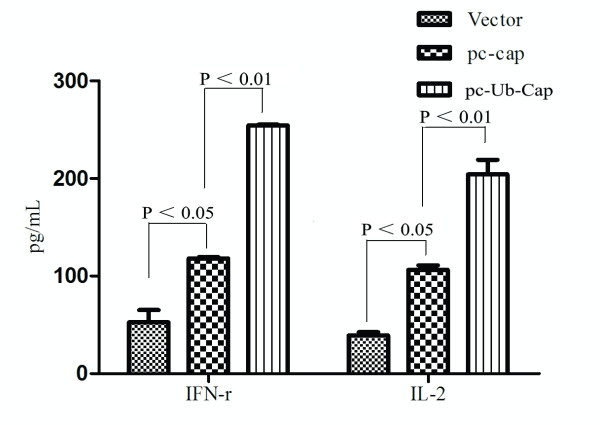
**Cytokine profile of proliferation T cells**. The splenocytes were re-stimulated with inactivated PCV2. The supernatants were collected. IFN-r and IL-2 concentration (ng/mL) were determined by ELISA.

To profile the cytokine levels induced by the DNA plasmids, an ELISA kit was used to measured levels of the Th1 cytokines, IFN-γ and IL-2, secreted by splenic lymphocyte cells six weeks after the last immunization. As depicted in Figure 4, the highest level of interferon-gamma (IFN-γ) and interleukin-2 (IL-2) was induced in the pc-Ub-Cap-immunized group, followed by the pc-Cap and vector-immunized groups.

### 3.6. Viremia after challenge

PCV2 DNA was detected by real-time PCR based on sequence coding for nucleocapsid protein from blood samples of PCV2 challenged mice. The occurrence of viremia and the mean number of PCV2 genomic copies are presented in Table 3. At weeks two, four, six and eight post challenge (p.c.), the viral load in the blood of mice immunized with DNA plasmids was less than in mice immunized with the empty vector. Specifically, the highest level of PCV2 DNA was observed in the vector-immunized group, followed by the pc-Cap and pc-Ub-Cap-immunized groups. The pc-Ub-Cap-immunized group exhibited a reduction in the level of viremia with a significantly decreased number of PCV2 genomic copies at four time points above (P < 0.05).

**Table 3:** Viraemia in mouse serum measured by quantitative PCR

group	No. of mice with viraemia/no. tested (mean lg PCV2 load ± SD) at the following weeks p.c.			
	**2**	**4**	**6**	**8**

Vector	7.56 ± 1.05^a^	7.12 ± 0.29a	6.27 ± 0.27^a^	5.77 ± 0.23^a^
pc-Cap	5.59 ± 0.51^b^	3.93 ± 0.18^b^	3.41 ± 0.51^b^	2.69 ± 0.33^b^
pc-Ub-Cap	3.97 ± 0.45^c^	3.00 ± 0.55^c^	2.45 ± 0.48^c^	0c

Shared letters above columns indicate no significant difference (P > 0.05), whilst different letters indicate significant differences among the groups (P < 0.05) (n = 5).

A load value of 0 is equal to < 10^2 ^copies ml^-1 ^(the threshold of sensitivity of the quantitative PCR).

## 4. Discussion

Previous studies reported that Cap protein induces protection against subsequent challenge with PCV2 and that ORF2 plasmid DNA vaccines [12,29-31] were more efficacious than Cap protein-based vaccines [12]. Additionally, two reports have shown that Ub-antigen conjugation can target endogenously synthesized antigenic proteins for rapid degradation and improve their antigenicity and immunogenicity [22,32]. Based on these reports, the pc-Ub-Cap was constructed and its ability to protect against PCV2 is promising.

To explore a more ideal DNA vaccine and enhance cell-mediated immune responses, we constructed a recombinant plasmid co-expressing swine ubiquitin and a Cap-encoding gene from PCV2. A Kozak consensus sequence was introduced upstream of ubiquitin, which is crucial for the efficient initiation of translation [33]. We mutated the C-terminal glycine residue of Ub to arginine as previous reported [34], because it is essential for a DNA vaccine to induce antigen-specific CD8+ T cells based on the Ub fusion degradation pathway.

PCV2-specific cellular immune responses were evaluated after restimulation in vitro with inactivated PCV2. Enhanced cell-mediated immune responses were observed in mice immunized with pc-Ub-Cap. In the present study, we investigated the Cap-specific lymphoproliferative activity, splenocyte phenotypes and levels of the Th1 cytokines, IFN-γ and IL-2, secreted by splenic lymphocytes induced by PCV2. The results demonstrated that the ubiquitin-fused DNA was more efficient in eliciting lymphoproliferative responses in CD4^+ ^T cells and CD8^+ ^T cells than the pc-Cap plasmid. Moreover, we found that the ubiquitin-fused antigen induced antigen-specific CD8^+ ^T cells based on the Ub fusion degradation pathway.

Th1 cells, which produce IL-2 and IFN-γ, induce the activation of macrophages, delayed-type hypersensitivity and the production of IgG2a [35,36]. When the immune profiles of mice at the stage of p. i. were explored, the Th1-associated cytokines (IFN-γ and IL-2) were expressed more by splenocytes from mice receiving DNA vaccines. Furthermore, in vivo studies showed that immunization with DNA plasmids induced greater IgG2a antibody production compared to IgG1, suggesting a stronger Th1 pattern of immune response. In our model, the pc-Ub-Cap-immunized mice exhibited higher levels Th1-based immune responses compared to the pc-Cap-vaccinated mice.

The IgG2a antibody is reportedly important in protecting against PCV2 infection [12]. This notion was confirmed by the viremia results from blood samples by quantitative, real-time PCR. Viremia was monitored at two, four, six and eight weeks p.c. by quantitative, real-time PCR. The results revealed that the viral loads in all vaccinated groups were significantly lower than the parental vector control, particularly at weeks eight p.c. At this time point, a high viremia was observed in the blood of the empty vector control group compared with a decreasing viremia in the blood of vaccinated animals. The viremia observed in each group was consistent with the total antibody response results by indirect ELISA assay, which demonstrated that mice immunized with pc-Ub-Cap developed significantly higher Cap-specific antibody titers than those inoculated with pc-Cap. Other reports showed that the total antibody response in PCV2 followed the NA titers in subclinically infected pigs, with seroconversion occurring from 15 dpi onwards [37,38]. Our results also suggest that the tendency of antibody to protect against PCV2 infection coincided with decrease of virus load.

## 5. Conclusions

In summary, we have characterized the immune response against PCV2 provided by the pc-Cap and the pc-Ub-Cap DNA vaccines in mice. In the present study, ubiquitin-tagging of the Cap antigen enhanced the Th1 profile of cellular immune response. In addition, a DNA plasmid fused with ubiquitin elicited a more robust humoral immune response than the plasmid without ubiquitin, suggesting that ubiquitin-fusion of an immunogen can improve its immunogenicity.

## 6. Competing interests

The authors declare that they have no competing interests.

## 7. Authors' contributions

FF carried out the molecular genetic studies, participated in the sequence alignment and drafted the manuscript. XuL carried out the immunoassays. YL participated in the sequence alignment and drafted the manuscript. YY participated in the design of the study and performed the statistical analysis. GT, GL, YJZ and XiL conceived of the study, and participated in its design and coordination and helped to draft the manuscript. All authors read and approved the final manuscript.
